# The Pro-inflammatory Effects of Glucocorticoids in the Brain

**DOI:** 10.3389/fendo.2016.00078

**Published:** 2016-06-28

**Authors:** Erica de Almeida Duque, Carolina Demarchi Munhoz

**Affiliations:** ^1^Department of Pharmacology, Institute of Biomedical Science, University of São Paulo, São Paulo, Brazil

**Keywords:** glucocorticoids, glucocorticoids receptors, brain, steroid hormones, inflammation, cytokines, pro-inflammatory

## Abstract

Glucocorticoids are a class of steroid hormones derived from cholesterol. Their actions are mediated by the glucocorticoid and mineralocorticoid receptors, members of the superfamily of nuclear receptors, which, once bound to their ligands, act as transcription factors that can directly modulate gene expression. Through protein–protein interactions with other transcription factors, they can also regulate the activity of many genes in a composite or tethering way. Rapid non-genomic signaling was also demonstrated since glucocorticoids can act through membrane receptors and activate signal transduction pathways, such as protein kinases cascades, to modulate other transcriptions factors and activate or repress various target genes. By all these different mechanisms, glucocorticoids regulate numerous important functions in a large variety of cells, not only in the peripheral organs but also in the central nervous system during development and adulthood. In general, glucocorticoids are considered anti-inflammatory and protective agents due to their ability to inhibit gene expression of pro-inflammatory mediators and other possible damaging molecules. Nonetheless, recent studies have uncovered situations in which these hormones can act as pro-inflammatory agents depending on the dose, chronicity of exposure, and the structure/organ analyzed. In this review, we will provide an overview of the conditions under which these phenomena occur, a discussion that will serve as a basis for exploring the mechanistic foundation of glucocorticoids pro-inflammatory gene regulation in the brain.

## Introduction

### Inflammation and Glucocorticoids

Inflammation is described as a response to infection or injury. The cells of innate immunity, such as neutrophils and macrophages in the periphery (1) and microglia (2) in the CNS, express receptors called pattern recognition receptors (PRRs) (3, 4) that are able to recognize parts of conserved evolutionarily microbial molecules called pathogen-associated molecular patterns (PAMPs) (5). They also can recognize endogenous molecules called alarmins or danger-associated molecular patterns (DAMPs), which are host-derived proteins released from cells following tissue injury (1, 6). It has been greatly demonstrated that chronic inflammation is associated with several pathologies, such as obesity (7), cardiovascular diseases (8), rheumatoid arthritis (9), inflammatory bowel disease (10, 11), asthma (12, 13), diabetes (14, 15), neurodegenerative diseases (16), and cancer (17). These diseases are mostly characterized by a dysregulated inflammatory response, when the normally protective role of inflammation becomes detrimental as the response becomes excessive in magnitude or duration.

Glucocorticoids (GCs, cortisol in primates and corticosterone – CORT – in rodents) are steroid hormones that essentially regulate a variety of physiological processes, including embryonic development, metabolism of carbohydrates, lipids, and proteins, the stress response, and the resolution of inflammation (6). GCs have potent anti-inflammatory and immunosuppressive action, reflecting the fact that they are the most widely prescribed drug for the treatment of a variety of inflammatory and autoimmune diseases (18). They are mainly synthetized in the suprarenal cortex as result of the activation of the hypothalamic–pituitary–adrenal axis (HPA axis).

In general, GCs are considered anti-inflammatory and protective agents, since they inhibit gene expression of pro-inflammatory mediators and other possible damaging molecules, e.g., reactive oxygen species (ROS), in peripheral organs and in the CNS. Despite the primary anti-inflammatory actions of GCs to resolve inflammatory process and restore homeostasis, it is becoming increasingly common studies, suggesting that GCs are not uniformly anti-inflammatory and in some cases may even increase certain parameters of the inflammatory response, mainly in the CNS (19), depending on the dose, chronicity of exposure, and structure/organ analyzed.

In this review, we will provide an overview of the conditions under which these phenomena occur, a discussion that will serve as a basis for exploring the mechanistic foundation of glucocorticoid potentiation of pro-inflammatory gene regulation.

### Glucocorticoid Receptor

As other steroids, GCs are lipophilic molecules that easily reach the cytoplasm of target cells *via* peripheral circulation, where they are bound to carrier proteins and then diffuse promptly through cell membranes to modulate gene transcription through their two different nuclear receptors: the mineralocorticoid receptor (MR) or the glucocorticoid receptor (GR) (20, 21). The affinity of CORT for MR is 10-fold higher than for GR, consequently, MR is heavily occupied by basal CORT levels, being mainly responsible for the physiological effects of GCs, and GR is only heavily occupied during pharmacological levels of CORT or during peak secretion of this hormone, commonly observed in stressful situations (22).

The GR molecular structure consists of three functional domains: (1) the N-terminal amino domain (NTD) that contains one of the main transactivation domain, called the activation function 1 (AF1). This domain is functionally important, since it is required for transcriptional activity of steroid hormones; (2) the DNA-binding domain (DBD), which binds to the glucocorticoid responsive elements (GRE) sites in the promoter region of target genes; and (3) ligand-binding domain (LBD) that contains the binding domain to glucocorticoids as well as important sequences for interaction with coregulators, whether coactivators and/or corepressors (23).

Two human isoforms of GR have been identified, GRα and GRβ, which are originated by alternative splicing of the primary transcript GR. The GRα is the predominant isoform of the receptor, and it is the one that transduces GCs signal (23). The GRβ differs from GRα in the carboxy terminal sequence, where the last 50 amino acids are replaced by a sequence of 15 non-homologous amino acids, making GRβ non-responsive to GCs (23, 24), with no transcription of target genes when GCs binds to GRβ through a mechanism involving the formation of heterodimers GRα–GRβ (23). The GRβ has been described in some cells, especially inflammatory cells, such as lymphocytes and macrophages, and related with GCs resistance in diseases, such as asthma (12) and chronic lymphocytic leukemia (25). Recently, GRβ was reported in liver of mice (26) and rats (27) with a role in metabolism control by insulin.

Glucocorticoid receptor isoforms are subject to various posttranslational alterations that further modulate receptor activity. After binding to GC, the conformational changes make them susceptible to modifications, i.e., phosphorylation, acetylation, ubiquitination, and sumoylation (23, 28–30), which influence DNA-binding complex force, affecting transcription and selection of genes regulated by this complex. The multiple phosphorylation events affecting GR NTD lead to changes in the spectrum of GR target genes and is thought to influence both transcriptional activity and nuclear trafficking (23, 30–32).

In conclusion, gene regulation by GCs is complex. The sequence of the DNA-binding site influences the conformation of the GC–GR, leading to different patterns of gene expression, including the pro-inflammatory ones. In addition, the recruitment of the GC–GR and their regulatory elements, as coactivators and RNA polymerase II to DNA binding, is also dependent on the existence of accessible chromatin, which appears to be cell type specific (29).

### Glucocorticoid Signal Transduction

Upon reaching the target tissue, GCs bind to nuclear receptors, classical GRs, in the cytoplasm (33). GR binding promotes their release from a multiprotein complex composed by chaperones, e.g., Hsp70, Hsp90, p23, and immunophilins (FKBP51, FKBP52) (18, 30), allowing the GC–GR complex to interact with other cytosolic proteins, or in most cases, to translocate to the nucleus as a result of the exposure of a nuclear localization domain (18). Upon ligation and translocation, GR dimerizes and binds to DNA at palindromic GRE sites, activating the promoter as a transcription factor (transactivation), altering gene expression of various proteins. The GRE structure is not exclusive for GR binding, as it is also common for other nuclear receptors, such as MR, progesterone, and androgen receptors. For that reason, it is referred from some authors as hormonal response elements (HREs) rather than GRE (34).

The GC–GR complex also acts without dimerization, as a monomeric complex, and binds to DNA together with other transcription factor, in this way cooperatively enhancing gene expression (composite transactivation). In addition, the GC–GR complex can also physically interact with transcription factors without interacting with DNA itself, thereby inhibiting the transcription factor and repressing transcription. This is the predominant transrepression mechanism and is called tethering; it is the mechanism responsible to inhibit the pro-inflammatory transcription factors, NFκB (35, 36) and activator protein 1 (AP-1) (37), and also other transcription factors, such as CREB (38), accounting for the anti-inflammatory and immunosuppressive GCs effects.

In addition to the GR-mediated genomic mechanisms, several rapid non-genomic pathways have also been reported. According to them, GCs can have direct effects on cell membrane, interacting with membrane GR (mGR), as reported in human lymphocytes and monocytes (39), and in skeletal muscle fibers in mice (40). GCs can also interact with classical GRs targeting signaling proteins, such as kinases, and/or with classic GRs translocating into the mitochondria, one of the probable mechanisms of apoptosis induced by GC–GR complex on T cells (41–43). These pathways can result in the induction of downstream signaling cascades, changes in cytoplasmic calcium, sodium, or potassium concentrations, and increase in mitochondrial production of ROS (21, 34, 44).

### The Pro-inflammatory Glucocorticoid Effects

The evidence supporting the pro-inflammatory GC effects is very robust and suggests that they occur in specific and very intricate situations. Timing, concentration, and duration of GC exposure are all critical in determining whether these hormones will be immunoactivator or immunosuppressor, but the specific situations leading to each outcome are not well documented.

Munhoz et al. (45, 46) have shown that chronic unpredictable stress (CUS), which chronically augment plasma CORT, and mid-to-high levels of CORT enhance the LPS-induced NFκB activation as well as the expression of pro-inflammatory genes (iNOS, IL-1β, TNF-α), while decrease the expression of classical anti-inflammatory ones (IL-1ra, IL-10, and MKP-1) in the frontal cortex and in a lesser extent in the hippocampus of rats. These pro-inflammatory GCs effects were region-dependent and GR-mediated, since they were not evident in the hypothalamus or heart of rats, and the pretreatment with the RU486 reversed them (45, 46).

Microglia are an important player in the GC endangerment to neurons. Sorrells et al. (47) have shown that the concurrent treatment with minocycline, a microglia inhibitor, but not indomethacin, a non-steroid anti-inflammatory drug, prevented GC endangerment in a model of neuronal death using kainic acid. High GC levels did not substantially affect the levels of the chemokines, CCL2, CINC-1, or baseline NFκB activity, but they did suppress mRNA levels of CX3CL1, CX3CR1, and CD22 in the hippocampus – factors that restrain inflammatory responses – while increased by twofold the IL-1β mRNA expression (47). In another study, using transgenic mice lacking GR expression in myeloid cells (microglia included), the same group showed that GC effects on myeloid cells have detrimental consequences for neuron survival in a kainic acid and transitory MCAO occlusion model (48). Indeed, GCs can activate microglia, putting them in a “primed” state (49–51). The primed microglia can undergo changes such as cell surface upregulation of receptors (e.g., TLRs and NLRs) (52, 53) and myeloid markers (e.g., MHC II) (54, 55), promoting a “sensitized” state without production of inflammatory or anti-inflammatory mediators, but, with further stimulus, can produce exaggerated levels of inflammatory mediators. It has been showed increased plasma levels of TNFα and IL-1β, and IL-1β content in various CNS regions (pituitary, hypothalamus, hippocampus, and cerebellum) shortly after the LPS challenge (56).

Timing is an important variable to determine the outcome of GCs actions. Frank et al. (50, 57) reported that CORT administered acutely before an LPS challenge facilitated the inflammatory response to the challenge, both in the periphery and in the brain. In contrast, CORT administered after the same immune challenge resulted in suppression of the inflammatory response (50). They further demonstrated that prior *in vivo* administration of CORT potentiated the pro-inflammatory response of isolated hippocampal microglia exposed, *ex vivo*, to LPS (51), establishing a direct relationship between CORT elevation and sensitizing hippocampal microglia.

Corticosterone is also important in the sensitization of microglia in aging. Aged rats, submitted to an immune challenge, show potentiated and longer inflammatory response. Such potentiation has been associated with impairments in hippocampal synaptic plasticity and in contextual and spatial forms of memory (57–59). Interestingly, these aged rats exhibited higher corticosterone levels in the hippocampus, but not in the plasma, throughout the diurnal inactive phase only. Furthermore, the elevated diurnal hippocampal CORT was associated with an increased expression of hippocampal 11β-hydroxysteroid dehydrogenase type 1 (11β-HSD1), the enzyme that catalyzes GCs inactive form, 1-dehydrocorticosterone, to the active one, CORT, and to a greater hippocampal GR activation, also only in the inactive phase of the day. Moreover, nuclear-specific expression of GR during the inactive phase was significantly higher in aged rats (59).

**Figure 1:**
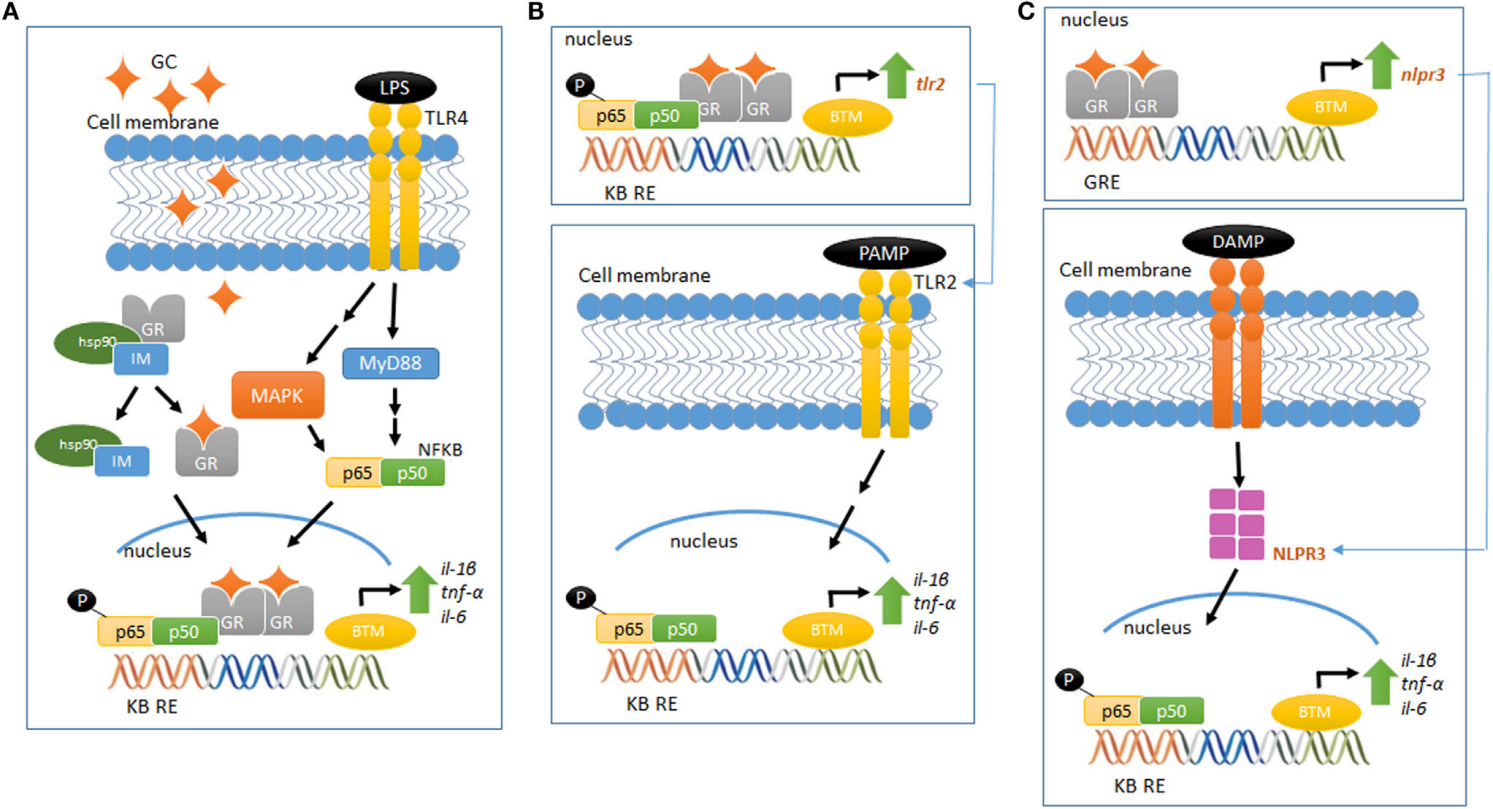
**Glucocorticoid-mediated modulation of pro-inflammatory pathways**. **(A)** LPS binds to TLR4 and activates the MAP kinase pathway, activating NFκB and increasing the gene expression of pro-inflammatory cytokines (IL-1β, TNF-α, and IL-6). Elevated and prolonged GC exposure increases and potentiates the pro-inflammatory response related to MAP kinase–NFκB pathway. **(B)** GR activation regulates the gene and protein expression of TLR2, which recognizes PAMPS. This event recruits intracellular proteins, ultimately leading to the downstream signaling activation of NFκB, increasing the expression of inflammatory cytokines, including IL-6, TNF-α, and IL-1β. **(C)** GR activation regulates the gene and protein expression of NLRP3, which recognizes DAMPS and regulates the immune system response in a mechanism similar to the proposed in **(B)**. Schematic figures based on Ref. (39, 70).

Although the molecular mechanism of GC pro-inflammatory actions is largely unknown, new players have been set in action. GCs upregulate the expression of the TLR2 and TLR4, both involved in the recognition of PAMPS and DAMPS and in the activation of signaling cascades that lead to the synthesis and release of inflammatory mediators (60). *In vitro* and *in vivo* studies have demonstrated that GCs can upregulate TLR2 expression by the activation of MAPK phosphatase-1 (MKP-1), which, in turn, inhibits p38 MAPK activity, a negative regulator for TLR2. The increased expression of TLR2 leads to enhanced cytokine expression, such as TNF-α, IL-1β, and IL-8, upon challenge with an inflammatory stimulus. In addition, TNF-α and GCs cooperate to stimulate the promoter for TLR2 and potentially TLR4, increasing receptor expression (57, 61, 62) (Figures 1A,B).

The scientific literature provides growing information about the recently described new class of intracellular PRRs, the nucleotide-binding domain, leucine-rich repeat-containing (NBD-LRR, referred as NLRs) (63). Of the several NLRs recognized, NLRP1, NLRP3, AIM2, and NLRC4 are able to assemble and form a multiprotein complex known as the inflammasome. This complex regulates the activation of caspase-1 and the subsequent maturation and release of pro-inflammatory cytokines, such as IL-1β and IL-18, in response to a wide variety of intracellular PRRs [for subject review, see Ref. (64)]. NLRP3 could be involved in GCs pro-inflammatory actions, as GCs potentiates the LPS-induced pro-inflammatory cytokine IL-1β (45, 46). Indeed, Busillo et al. (65) demonstrated that NLRP3 is a GC-responsive gene in macrophages, an effect GR-dependent. Glucocorticoid induction of NLRP3 sensitizes macrophages to extracellular ATP, which results in the secretion of pro-inflammatory cytokines, such as IL-1β, TNF-α, and IL-6 (65). Furthermore, GCs have been suggested to induce the expression of the purinergic receptor P2Y2R, which once activated, enhances IL-6 secretion by endothelial cells in response to ATP (66). Therefore, GC-mediated activation of TLR2, NLRP3, P2Y2R, and potentiation of TNF-α- and pro-inflammatory genes provide a potential mechanism by which these hormones exert pro-inflammatory actions (Figure 1C).

In addition, Weber and colleagues reported a new stress-induced pro-inflammatory mediator, the alarmin high-mobility group box-1 protein (HMGB-1) (67). HMGB-1 has been reported to be present in the CNS and to play a role in mediating neuroinflammatory responses to ischemia and other injuries, such as immune disorders and neurodegenerative diseases [extensively reviewed in Ref. (68)]. Severe acute stress increases HMGB-1 protein levels in the hippocampus, specifically in the cytosol of hippocampal microglia isolated from severely acute stressed rats. NLRP3, IKBα, and NFKB proteins levels are also increased immediately after severe acute stress. Interestingly, Box A (a HMGB-1 antagonist) pretreatment blocked the stress-induced increase in microglial IL-1β, IKBα, and NLRP3 mRNA expression in response to a bacterial challenge (67). These results suggest that HMGB-1 participates in stress-inducing NLRP3 priming, adding another mechanism by which GCs could have pro-inflammatory effects in the brain (67, 69).

## Mineralocorticoids

Aldosterone is the steroidal hormone produced in the cortex of suprarenal gland that specifically binds to the MR. Although aldosterone is the related ligand of the MR, GCs can also bind to this receptor with equivalent affinity. The production and secretion of this hormone are majorly triggered in response to alterations in blood perfusion, perceived by cells in the juxtaglomerular complex. The main goal of this action is to maintain the blood pressure in a normal range in terms of control of water and electrolyte homeostasis (70). Mineralocorticoids can also modulate inflammation. MR expression is observed in cells of the immune system and aldosterone has been associated with pro-inflammatory immune effects, such as the release of pro-inflammatory cytokines, generating oxidative stress and inducing fibrosis (71).

Clinical studies have demonstrated that MR antagonism in cardiovascular diseases can blunt inflammatory damage, an important step in the induction of cardiac fibrosis (72). *In vitro* studies, using myeloid murine cells, have suggested an important role of mineralocorticoid in inducing changes in macrophages phenotype and, therefore, function.

In response to different inflammatory stimuli, macrophages can undergo classical M1 activation (stimulated by TLR ligands and IFN-γ) or alternative M2 activation (stimulated by IL-4/IL-13 cytokines); these states would correspond to the Th1–Th2 polarization of T cells [for review, see Ref. (73)]. In this sense, in dendritic cells from mice, it was shown that aldosterone induces the secretion of IL-6 and TGF-β, two pro-inflammatory cytokines necessary for the polarization of the adaptive immune response toward a Th17 phenotype, proning the system to autoimmunity (74). In peritoneal macrophages, aldosterone treatment promoted the activation of M1 macrophages, inducing TNFα, MCP-1, and IL12 gene expression, all those prevented by MR antagonism (75).

Regarding molecular mechanisms, it has been suggested that aldosterone, through MR, could regulate the expression of several genes. Indeed, cytokine promoters contain HREs, which can be modulated by GCs and potentially by mineralocorticoids (20). Despite it was suggested that ligand–receptor binding to simple HRE is enough to induce gene expression, many other proteins are able to modulate these interactions, adding specificity to gene transcription induced by MR. Also, this interaction could be more complex once the HRE also contains binding sites for non-receptor factors, the composite HREs (76).

In the brain, MR activation is important to maintain many functions of this tissue [for review, see Ref. (77)]. Under pathological conditions, such as cerebral stroke, MR antagonism or ablation has been associated with reduction of ischemic area, lower markers of remodeling process in cerebral vessels, and reduction of microglial/macrophages infiltration and activation, with decreased expression of cytokines and chemokines, such as IL-1β, IL-6, TNF-α, MCP-1, and MIP-1. It was also reported that the MR activation in myeloid cells exacerbates inflammation and alters the M1/M2 inflammatory response to stroke (78). Finally, a study using microglial cells BV-2 reported that the stimulation with aldosterone resulted in NFκB activation and the nuclear translocation of its p65 subunit, further upregulating the expression of IL-6 and TNFr2, reinforcing the evidence of pro-inflammatory actions of mineralocorticoids in the brain (79).

## Concluding Remarks

Together, these findings suggest that exposure to steroid hormones in special conditions can shift the (neuro)immune microenvironment toward a pro-inflammatory state, predisposing certain regions of the CNS to a stronger pro-inflammatory response, if the organism is exposed to a subsequent challenge. Given the clinical implications of the pro-inflammatory steroid hormones actions, it is important to emphasize the need for continued basic investigations to understand the unexpected capacity of these hormones to increase CNS inflammation. Future research must be directed at determining how steroid hormones act on different cell types and regions to produce such markedly different immunomodulatory outcomes.

## Author Contributions

ED made substantial intellectual contributions to the conception, design, interpretation, drafting, and revising of the work, critically. CM made substantial contributions to conception, design, interpretation; revision critically for important intellectual content; and final approval of the version to be published.

## Conflict of Interest Statement

The authors declare that the research was conducted in the absence of any commercial or financial relationships that could be construed as a potential conflict of interest.

## References

[1] Medzhitov R Origin and physiological roles of inflammation. Nature (2008) 454(7203):428–35. 10.1038/nature07201 18650913

[2] Saijo K Glass CK. Microglial cell origin and phenotypes in health and disease. Nat Rev Immunol (2011) 11(11):775–87. 10.1038/nri3086 22025055

[3] Palm NW Medzhitov R. Pattern recognition receptors and control of adaptive immunity. Immunol Rev (2009) 227(1):221–33. 10.1111/j.1600-065X.2008.00731.x 19120487

[4] Akira S. Innate immunity and adjuvants. Philos Trans R Soc Lond B Biol Sci (2011) 366(1579):2748–55. 10.1098/rstb.2011.0106 21893536 PMC3146784

[5] Strowig T Henao-Mejia J Elinav E Flavell R. Inflammasomes in health and disease. Nature (2012) 481(7381):278–86. 10.1038/nature10759 22258606

[6] Medzhitov R. Inflammation 2010: new adventures of an old flame. Cell (2010) 140(6):771–6. 10.1016/j.cell.2010.03.006 20303867

[7] Wensveen FM Jelencic V Valentic S Sestan M Wensveen TT Theurich S NK cells link obesity-induced adipose stress to inflammation and insulin resistance. Nat Immunol (2015) 16(4):376–85. 10.1038/ni.3120 25729921

[8] Golia E Limongelli G Natale F Fimiani F Maddaloni V Pariggiano I Inflammation and cardiovascular disease: from pathogenesis to therapeutic target. Curr Atheroscler Rep (2014) 16(9):435. 10.1007/s11883-014-0435-z 25037581

[9] Muller-Ladner U Pap T Gay RE Neidhart M Gay S. Mechanisms of disease: the molecular and cellular basis of joint destruction in rheumatoid arthritis. Nat Clin Pract Rheumatol (2005) 1(2):102–10. 10.1038/ncprheum0047 16932639

[10] Baumgart DC Sandborn WJ. Crohn’s disease. Lancet (2012) 380(9853):1590–605. 10.1016/S0140-6736(12)60026-9 22914295

[11] Pedersen J LaCasse EC Seidelin JB Coskun M Nielsen OH. Inhibitors of apoptosis (IAPs) regulate intestinal immunity and inflammatory bowel disease (IBD) inflammation. Trends Mol Med (2014) 20(11):652–65. 10.1016/j.molmed.2014.09.006 25282548

[12] Sousa AR Lane SJ Cidlowski JA Staynov DZ Lee TH. Glucocorticoid resistance in asthma is associated with elevated in vivo expression of the glucocorticoid receptor beta-isoform. J Allergy Clin Immunol (2000) 105(5):943–50. 10.1067/mai.2000.106486 10808175

[13] Wenzel S. Severe asthma: from characteristics to phenotypes to endotypes. Clin Exp Allergy (2012) 42(5):650–8. 10.1111/j.1365-2222.2011.03929.x 22251060

[14] Donath MY Shoelson SE. Type 2 diabetes as an inflammatory disease. Nat Rev Immunol (2011) 11(2):98–107. 10.1038/nri2925 21233852

[15] Esser N Paquot N Scheen AJ. Anti-inflammatory agents to treat or prevent type 2 diabetes, metabolic syndrome and cardiovascular disease. Expert Opin Investig Drugs (2015) 24(3):283–307. 10.1517/13543784.2015.974804 25345753

[16] Glass CK Saijo K Winner B Marchetto MC Gage FH. Mechanisms underlying inflammation in neurodegeneration. Cell (2010) 140(6):918–34. 10.1016/j.cell.2010.02.016 20303880 PMC2873093

[17] Colotta F Allavena P Sica A Garlanda C Mantovani A. Cancer-related inflammation, the seventh hallmark of cancer: links to genetic instability. Carcinogenesis (2009) 30(7):1073–81. 10.1093/carcin/bgp127 19468060

[18] Schweingruber N Reichardt SD Luhder F Reichardt HM. Mechanisms of glucocorticoids in the control of neuroinflammation. J Neuroendocrinol (2012) 24(1):174–82. 10.1111/j.1365-2826.2011.02161.x 21615563

[19] Sorrells SF Caso JR Munhoz CD Sapolsky RM. The stressed CNS: when glucocorticoids aggravate inflammation. Neuron (2009) 64(1):33–9. 10.1016/j.neuron.2009.09.032 19840546 PMC4782919

[20] Beato M Klug J. Steroid hormone receptors: an update. Hum Reprod Update (2000) 6(3):225–36. 10.1093/humupd/6.3.225 10874567

[21] Sakamoto H Takahashi H Matsuda K Nishi M Takanami K Ogoshi M Rapid signaling of steroid hormones in the vertebrate nervous system. Front Biosci (Landmark Ed) (2012) 17:996–1019. 10.2741/3970 22201787

[22] McEwen BS Biron CA Brunson KW Bulloch K Chambers WH Dhabhar FS The role of adrenocorticoids as modulators of immune function in health and disease: neural, endocrine and immune interactions. Brain Res Brain Res Rev (1997) 23(1–2):79–133. 10.1016/S0165-0173(96)00012-4 9063588

[23] Oakley RH Cidlowski JA. Cellular processing of the glucocorticoid receptor gene and protein: new mechanisms for generating tissue-specific actions of glucocorticoids. J Biol Chem (2011) 286(5):3177–84. 10.1074/jbc.R110.179325 21149445 PMC3030321

[24] Ligr M Li Y Logan SK Taneja S Melamed J Lepor H Mifepristone inhibits GRbeta coupled prostate cancer cell proliferation. J Urol (2012) 188(3):981–8. 10.1016/j.juro.2012.04.102 22819113 PMC3646901

[25] Shahidi H Vottero A Stratakis CA Taymans SE Karl M Longui CA Imbalanced expression of the glucocorticoid receptor isoforms in cultured lymphocytes from a patient with systemic glucocorticoid resistance and chronic lymphocytic leukemia. Biochem Biophys Res Commun (1999) 254(3):559–65. 10.1006/bbrc.1998.9980 9920778

[26] Hinds TD Jr Ramakrishnan S Cash HA Stechschulte LA Heinrich G Najjar SM Discovery of glucocorticoid receptor-beta in mice with a role in metabolism. Mol Endocrinol (2010) 24(9):1715–27. 10.1210/me.2009-0411me.2009-0411 20660300 PMC2940475

[27] DuBois DC Sukumaran S Jusko WJ Almon RR. Evidence for a glucocorticoid receptor beta splice variant in the rat and its physiological regulation in liver. Steroids (2013) 78(2):312–20. 10.1016/j.steroids.2012.11.014 23257260 PMC3552070

[28] Meijsing SH Pufall MA So AY Bates DL Chen L Yamamoto KR. DNA binding site sequence directs glucocorticoid receptor structure and activity. Science (2009) 324(5925):407–10. 10.1126/science.1164265 19372434 PMC2777810

[29] John S Sabo PJ Thurman RE Sung MH Biddie SC Johnson TA Chromatin accessibility pre-determines glucocorticoid receptor binding patterns. Nat Genet (2011) 43(3):264–8. 10.1038/ng.759 21258342 PMC6386452

[30] Oakley RH Cidlowski JA. The biology of the glucocorticoid receptor: new signaling mechanisms in health and disease. J Allergy Clin Immunol (2013) 132(5):1033–44. 10.1016/j.jaci.2013.09.007 24084075 PMC4084612

[31] Ratman D Vanden Berghe W Dejager L Libert C Tavernier J Beck IM How glucocorticoid receptors modulate the activity of other transcription factors: a scope beyond tethering. Mol Cell Endocrinol (2013) 380(1–2):41–54. 10.1016/j.mce.2012.12.014 23267834

[32] Carruthers CW Suh JH Gustafsson JA Webb P. Phosphorylation of glucocorticoid receptor tau1c transactivation domain enhances binding to CREB binding protein (CBP) TAZ2. Biochem Biophys Res Commun (2015) 457(1):119–23. 10.1016/j.bbrc.2014.12.021 25511704

[33] Li MD Yang X. A retrospective on nuclear receptor regulation of inflammation: lessons from GR and PPARs. PPAR Res (2011) 2011:742785. 10.1155/2011/742785 21941526 PMC3175381

[34] Falkenstein E Tillmann HC Christ M Feuring M Wehling M Multiple actions of steroid hormones – a focus on rapid, nongenomic effects. Pharmacol Rev (2000) 52(4):513–56. 11121509

[35] De Bosscher K Vanden Berghe W Haegeman G. Cross-talk between nuclear receptors and nuclear factor kappaB. Oncogene (2006) 25(51):6868–86. 10.1038/sj.onc.1209935 17072333

[36] Beck IM Vanden Berghe W Vermeulen L Yamamoto KR Haegeman G De Bosscher K. Crosstalk in inflammation: the interplay of glucocorticoid receptor-based mechanisms and kinases and phosphatases. Endocr Rev (2009) 30(7):830–82. 10.1210/er.2009-0013 19890091 PMC2818158

[37] Karin M Chang L AP-1 – glucocorticoid receptor crosstalk taken to a higher level. J Endocrinol (2001) 169(3):447–51. 10.1677/joe.0.1690447 11375114

[38] Whitehead D Carter DA. cAMP response element-binding protein phosphorylation and DNA binding activity are increased in the anterior pituitary gland following glucocorticoid depletion. J Mol Endocrinol (1997) 19(3):291–7. 10.1677/jme.0.0190291 9460650

[39] Bartholome B Spies CM Gaber T Schuchmann S Berki T Kunkel D Membrane glucocorticoid receptors (mGCR) are expressed in normal human peripheral blood mononuclear cells and up-regulated after in vitro stimulation and in patients with rheumatoid arthritis. FASEB J (2004) 18(1):70–80. 10.1096/fj.03-0328com 14718388

[40] Boncompagni S Arthurton L Akujuru E Pearson T Steverding D Protasi F Membrane glucocorticoid receptors are localised in the extracellular matrix and signal through the MAPK pathway in mammalian skeletal muscle fibres. J Physiol (2015) 593(12):2679–92. 10.1113/JP270502 25846902 PMC4500352

[41] Rhen T Cidlowski JA Antiinflammatory action of glucocorticoids – new mechanisms for old drugs. N Engl J Med (2005) 353(16):1711–23. 10.1056/NEJMra050541 16236742

[42] Sionov RV Cohen O Kfir S Zilberman Y Yefenof E. Role of mitochondrial glucocorticoid receptor in glucocorticoid-induced apoptosis. J Exp Med (2006) 203(1):189–201. 10.1084/jem.20050433 16390935 PMC2118093

[43] Sundahl N Bridelance J Libert C De Bosscher K Beck IM. Selective glucocorticoid receptor modulation: new directions with non-steroidal scaffolds. Pharmacol Ther (2015) 152:28–41. 10.1016/j.pharmthera.2015.05.001 25958032

[44] Smith LK Cidlowski JA. Glucocorticoid-induced apoptosis of healthy and malignant lymphocytes. Prog Brain Res (2010) 182:1–30. 10.1016/S0079-6123(10)82001-1 20541659 PMC4770454

[45] Munhoz CD Lepsch LB Kawamoto EM Malta MB Lima Lde S Avellar MC Chronic unpredictable stress exacerbates lipopolysaccharide-induced activation of nuclear factor-kappaB in the frontal cortex and hippocampus via glucocorticoid secretion. J Neurosci (2006) 26(14):3813–20. 10.1523/JNEUROSCI.4398-05.2006 16597735 PMC6674142

[46] Munhoz CD Sorrells SF Caso JR Scavone C Sapolsky RM. Glucocorticoids exacerbate lipopolysaccharide-induced signaling in the frontal cortex and hippocampus in a dose-dependent manner. J Neurosci (2010) 30(41):13690–8. 10.1523/JNEUROSCI.0303-09.2010 20943909 PMC3842494

[47] Sorrells SF Munhoz CD Manley NC Yen S Sapolsky RM. Glucocorticoids increase excitotoxic injury and inflammation in the hippocampus of adult male rats. Neuroendocrinology (2014) 100(2–3):129–40. 10.1159/000367849 25228100 PMC4304880

[48] Sorrells SF Caso JR Munhoz CD Hu CK Tran KV Miguel ZD Glucocorticoid signaling in myeloid cells worsens acute CNS injury and inflammation. J Neurosci (2013) 33(18):7877–89. 10.1523/JNEUROSCI.4705-12.2013 23637179 PMC3691990

[49] Frank MG Baratta MV Sprunger DB Watkins LR Maier SF. Microglia serve as a neuroimmune substrate for stress-induced potentiation of CNS pro-inflammatory cytokine responses. Brain Behav Immun (2007) 21(1):47–59. 10.1016/j.bbi.2006.03.005 16647243 PMC13674783

[50] Frank MG Miguel ZD Watkins LR Maier SF Prior exposure to glucocorticoids sensitizes the neuroinflammatory and peripheral inflammatory responses to *E. coli* lipopolysaccharide. Brain Behav Immun (2010) 24(1):19–30. 10.1016/j.bbi.2009.07.008 19647070

[51] Frank MG Thompson BM Watkins LR Maier SF. Glucocorticoids mediate stress-induced priming of microglial pro-inflammatory responses. Brain Behav Immun (2012) 26(2):337–45. 10.1016/j.bbi.2011.10.005 22041296 PMC5652300

[52] Lehnardt S Massillon L Follett P Jensen FE Ratan R Rosenberg PA Activation of innate immunity in the CNS triggers neurodegeneration through a toll-like receptor 4-dependent pathway. Proc Natl Acad Sci U S A (2003) 100(14):8514–9. 10.1073/pnas.1432609100 12824464 PMC166260

[53] Lehnardt S. Innate immunity and neuroinflammation in the CNS: the role of microglia in toll-like receptor-mediated neuronal injury. Glia (2010) 58(3):253–63. 10.1002/glia.20928 19705460

[54] Juedes AE Ruddle NH. Resident and infiltrating central nervous system APCs regulate the emergence and resolution of experimental autoimmune encephalomyelitis. J Immunol (2001) 166(8):5168–75. 10.4049/jimmunol.166.8.5168 11290800

[55] Olson JK Miller SD. Microglia initiate central nervous system innate and adaptive immune responses through multiple TLRs. J Immunol (2004) 173(6):3916–24. 10.4049/jimmunol.173.6.3916 15356140

[56] Johnson JD O’Connor KA Deak T Stark M Watkins LR Maier SF. Prior stressor exposure sensitizes LPS-induced cytokine production. Brain Behav Immun (2002) 16(4):461–76. 10.1006/brbi.2001.0638 12096891

[57] Frank MG Barrientos RM Watkins LR Maier SF Aging sensitizes rapidly isolated hippocampal microglia to LPS ex vivo. J Neuroimmunol (2010) 226(1–2):181–4. 10.1016/j.jneuroim.2010.05.022 20537730 PMC2937085

[58] Chapman TR Barrientos RM Ahrendsen JT Maier SF Patterson SL. Synaptic correlates of increased cognitive vulnerability with aging: peripheral immune challenge and aging interact to disrupt theta-burst late-phase long-term potentiation in hippocampal area CA1. J Neurosci (2010) 30(22):7598–603. 10.1523/JNEUROSCI.5172-09.2010 20519534 PMC2891807

[59] Barrientos RM Thompson VM Kitt MM Amat J Hale MW Frank MG Greater glucocorticoid receptor activation in hippocampus of aged rats sensitizes microglia. Neurobiol Aging (2015) 36(3):1483–95. 10.1016/j.neurobiolaging.2014.12.003 25559333 PMC4346455

[60] Weber MD Frank MG Sobesky JL Watkins LR Maier SF. Blocking toll-like receptor 2 and 4 signaling during a stressor prevents stress-induced priming of neuroinflammatory responses to a subsequent immune challenge. Brain Behav Immun (2013) 32:112–21. 10.1016/j.bbi.2013.03.004 23500798 PMC3810175

[61] Hermoso MA Matsuguchi T Smoak K Cidlowski JA. Glucocorticoids and tumor necrosis factor alpha cooperatively regulate toll-like receptor 2 gene expression. Mol Cell Biol (2004) 24(11):4743–56. 10.1128/MCB.24.11.4743-4756.2004 15143169 PMC416411

[62] Wohleb ES Hanke ML Corona AW Powell ND Stiner LM Bailey MT beta-Adrenergic receptor antagonism prevents anxiety-like behavior and microglial reactivity induced by repeated social defeat. J Neurosci (2011) 31(17):6277–88. 10.1523/JNEUROSCI.0450-11.2011 21525267 PMC3160240

[63] Meylan E Tschopp J Karin M. Intracellular pattern recognition receptors in the host response. Nature (2006) 442(7098):39–44. 10.1038/nature04946 16823444

[64] Man SM Kanneganti TD. Regulation of inflammasome activation. Immunol Rev (2015) 265(1):6–21. 10.1111/imr.12296 25879280 PMC4400844

[65] Busillo JM Azzam KM Cidlowski JA. Glucocorticoids sensitize the innate immune system through regulation of the NLRP3 inflammasome. J Biol Chem (2011) 286(44):38703–13. 10.1074/jbc.M111.275370 21940629 PMC3207479

[66] Ding Y Gao ZG Jacobson KA Suffredini AF. Dexamethasone enhances ATP-induced inflammatory responses in endothelial cells. J Pharmacol Exp Ther (2010) 335(3):693–702. 10.1124/jpet.110.171975 20826566 PMC2993554

[67] Weber MD Frank MG Tracey KJ Watkins LR Maier SF. Stress induces the danger-associated molecular pattern HMGB-1 in the hippocampus of male Sprague Dawley rats: a priming stimulus of microglia and the NLRP3 inflammasome. J Neurosci (2015) 35(1):316–24. 10.1523/JNEUROSCI.3561-14.2015 25568124 PMC4287150

[68] Kang R Chen R Zhang Q Hou W Wu S Cao L HMGB1 in health and disease. Mol Aspects Med (2014) 40:1–116. 10.1016/j.mam.2014.05.001 25010388 PMC4254084

[69] van Zoelen MA Yang H Florquin S Meijers JC Akira S Arnold B Role of toll-like receptors 2 and 4, and the receptor for advanced glycation end products in high-mobility group box 1-induced inflammation in vivo. Shock (2009) 31(3):280–4. 10.1097/SHK.0b013e318186262d 19218854 PMC4535325

[70] Briet M Schiffrin EL. Aldosterone: effects on the kidney and cardiovascular system. Nat Rev Nephrol (2010) 6(5):261–73. 10.1038/nrneph.2010.30 20234356

[71] Azibani F Fazal L Chatziantoniou C Samuel JL Delcayre C. Aldosterone mediates cardiac fibrosis in the setting of hypertension. Curr Hypertens Rep (2013) 15(4):395–400. 10.1007/s11906-013-0354-3 23686824

[72] Rossignol P Menard J Fay R Gustafsson F Pitt B Zannad F. Eplerenone survival benefits in heart failure patients post-myocardial infarction are independent from its diuretic and potassium-sparing effects. Insights from an EPHESUS (eplerenone post-acute myocardial infarction heart failure efficacy and survival study) substudy. J Am Coll Cardiol (2011) 58(19):1958–66. 10.1016/j.jacc.2011.04.049 22032706

[73] Sica A Mantovani A. Macrophage plasticity and polarization: in vivo veritas. J Clin Invest (2012) 122(3):787–95. 10.1172/JCI59643 22378047 PMC3287223

[74] Herrada AA Contreras FJ Marini NP Amador CA Gonzalez PA Cortes CM Aldosterone promotes autoimmune damage by enhancing Th17-mediated immunity. J Immunol (2010) 184(1):191–202. 10.4049/jimmunol.0802886jimmunol.0802886 19949098

[75] Usher MG Duan SZ Ivaschenko CY Frieler RA Berger S Schutz G Myeloid mineralocorticoid receptor controls macrophage polarization and cardiovascular hypertrophy and remodeling in mice. J Clin Invest (2010) 120(9):3350–64. 10.1172/JCI41080 20697155 PMC2929712

[76] Cruz-Topete D Cidlowski JA. One hormone, two actions: anti- and pro-inflammatory effects of glucocorticoids. Neuroimmunomodulation (2015) 22(1–2):20–32. 10.1159/000362724 25227506 PMC4243162

[77] Reul JM Gesing A Droste S Stec IS Weber A Bachmann C The brain mineralocorticoid receptor: greedy for ligand, mysterious in function. Eur J Pharmacol (2000) 405(1–3):235–49. 10.1016/S0014-2999(00)00677-4 11033331

[78] Frieler RA Meng H Duan SZ Berger S Schutz G He Y Myeloid-specific deletion of the mineralocorticoid receptor reduces infarct volume and alters inflammation during cerebral ischemia. Stroke (2011) 42(1):179–85. 10.1161/STROKEAHA.110.598441 21106954 PMC3042719

[79] Chantong B Kratschmar DV Nashev LG Balazs Z Odermatt A. Mineralocorticoid and glucocorticoid receptors differentially regulate NF-kappaB activity and pro-inflammatory cytokine production in murine BV-2 microglial cells. J Neuroinflammation (2012) 9:260. 10.1186/1742-2094-9-260 23190711 PMC3526453

